# Supplementary material for: Snack Food Consumption across the Pune Transect in India: A Comparison of Dietary Behaviors Based on Consumer Characteristics and Locations
Source: Nutrients. 2021 Nov 30;13(12):4325. doi: 10.3390/nu13124325 (PMC8707810; doi:10.3390/nu13124325)
Supplement: Supplementary file 1 [file nutrients-13-04325-s001.zip › nutrients-1455325-supplementary.pdf]

Study Title:

Snack Food consumption pattern in Pune

Sr, No-
Date of Interview-
Interview start time-
Interviewer Name (Only trained staff)
Place of data collection- Rural/Urban/ Urban Slum
Age group- Children/ Adolescents/ adults
Sex- M/F

Tick the completed Questionnaires

1) Socio-demographic
2) consumption pattern
3) Physical activity
4) Snacking behavior
5) Anthropometric
6) Household Questionnaire
7) Focused Group Discussion

1) Socio-Demographic Questionnaire

1) Name of the respondent
2) Are you head of the family?
3) What is your mobile no?

Sr No	Name	Sex	Age	Education	Marital status	occupation	Place of work	Migration

4) Religion of the respondent

Hindu/ Islamic/ Christian / Sikh/ Buddhist/ Others, specify

5) Social Group of the respondent

Scheduled caste/ Scheduled Tribe/ Other Backward caste/ Open category/ Others, specify

6) What is the primary occupation of respondent (family members?)

1	Agriculture & allied activities
2	Share cropper/tenant
3	Wage labor (farm/non farm)
4	Primary processing (food processing, small industry, handicrafts, etc)
5	Trade and business (trader, shopkeeper, tailor, blacksmith, carpenter, etc)
6	Professional (doctor, engineer, lawyer, teacher, etc)
7	Govt employee/semi govt/political office bearer
8	Service
9	Self employed
10	No occupation

7) What is your average monthly expenditure

8) What is your average monthly expenditure on food items in Rupee?

9) How many people currently live in this household (including you)?

10) Do you have a ration card? 1. Yes 2. No

11) If yes, what is the color of your ration card? 1) White 2) Yellow 3) Orange

12) Do you have Aadhar Card? 1. Yes 2. No

13) Do you have Bank account? 1. Yes 2. No

14] Source of drinking water piped water/ well/ hand pump/ tap water/ other

15) Do you own any land? 1. Yes 2. No

16) If yes, how much? (Acre/)

17) If yes, how much of it is irrigated? (Acre)

	What crops do you grow?	How much do you consume in the house?/ Does any of the farm food consumption come from the farms?
1 Cereals (rice + wheat)		
2 Coarse cereals (maize, sorghum, pearl millet, ragi)		
3 Fruits		
4 Vegetables		
5 Cotton/sericulture		
6 Pulses		
7 Oilseeds		
8 Sugarcane		
9 Dairy		
10 Poultry		
11 Roots/tubers		
12 Others		

18) What assets does your household possess?

1 Electricity Connection
2 Ceiling Fan
3 LPG gas
4 Two wheelers including motor cycle
5 Color TV
6 Fridge
7 Washing Machine
8 PC /laptop
9 Car, Jeep, Van
10 Air Conditioner
11 Agriculture Land

2) Food frequency questionnaire

What kind of diet you prefer?

a) Vegetarian / b) Non-vegetarian

**Frequency of consumption daily/ weekly/ monthly and average serve consumed at a time**	**Frequency D/W/M**	**Number**	**Average Size S/M/L**	**Source Homemade Outside**	**Tentative Weekly/ monthly Expenses (Rs.**
1. Roti					
2. Rice					
3. Pulses/Dals					
4. Other Vegetables					
5. Other Fruits					
6. Vitamin A rich Fruits and Vegetables					
7. Vitamin C rich Fruits and Vegetables					
8. Green leafy vegetables					
9. Milk and Milk products (low fat) (Milk, Curd, Buttermilk, Paneer)					
10. Eggs					
11. Flesh foods (Meat, Fish, Poultry, Red meat)					
12. Protein powder/ Supplements					
13. Beverages (Tea/Coffee)					
14. Homemade snacks (Breakfast snacks- Pohe, Upma)					
15. Homemade fried snacks (Chakli, Shankar pale, Chiwda)					
16. Homemade sweet snacks (Laddu, Kheer, Basundi, Puranpoli)					
17. Bakery products (Pattice, Khari, Nankatai)					
18. Outside packaged salty snacks (Bobby, Chips, Kurkure)					
19. Outside fried snacks (Batata wada, Samosa...)					
20. Outside sweet Snacks (Mithai, Ice-cream, milkshakes…)					
21. Chocolates (Jelly sweets, Cadbury)					
22. Outside Chinese/ Punjabi food/ fast foods (Pav Bhaji, Pizza)					
23. Biscuits					
24. Sugar-sweetened beverages (Aerated drinks…)					
25. Fruit juice					

3) Physical Activity Questionnaire

Activity	Type	Time in Min./ day
**In house**	Sedentary Activities (self-care/cooking, sweeping. Swabbing, cleaning)	
	Moderate Activities (hand grinding/ washing clothes with hand)	
	Heavy Activities (Fetching water from well)	
**At work**	Not applicable	
	Sedentary Activities (Students/ Table jobs / IT professionals)	
	Moderate Activities (Farming/ Mechanic)	
	Heavy Activities (construction worker)	
**Outdoor game**	Not applicable	
	Sedentary Activities (walking/ Yoga)	
	Moderate Activities (running/ skipping rope/ climbing hill)	
	Heavy Activities (Competitive sports/ Talim/ swimming/ weight training)	
**Recreational**	Screen time (television, mobile)	
	Other (reading/ painting)	
	Watching movies	**(time/week)**
**Traveling**	Not applicable	
	Sedentary Activities (Two-wheeler/ Bus/ Train)	
	Moderate Activities (Walking/ Cycling)	
**Sleeping**	Day time	
	Night	
	Weekends	**(time/week)**
**Total minutes spent in all activities/day should not exceed 1440 mins**		

4) Snacking behavior assessment questionnaire

1) How many meals out of following do you have in a day?
a) Breakfast b) Lunch c) Dinner
2) Do you eat snack in between a) Yes b) No
3) What time do you generally have snack?
a) Morning b) Afternoon c) evening d) midnight e) late-night
If yes_____ from where you get snacks?
a) Take from home b) Buy from shop/ canteen/ outside

4) What are your favorite snacks?

	Home made/ outside
a) Fried snacks- Chips/samosa/ vada/ bhaji\	
b) Salty snacks- namkeen/ farsan	
c) Carbonated soft drinks	
d) Bakery products- bread/ cakes/ biscuits/ khari/	
e) Chocolates & Candies	
f) Fresh fruits/ Salad/ nuts/ seeds/ low fat dairy products	
g) Home made break fast snacks pohe/upma	
h) Home made fried snacks- Chiwda/ chakli/ laddoo/ /	
e) Others- fruits/dry fruits	

5) What activity is most common while having snack?
a) While watching TV
b) After school/ college while going home
c) While hanging out with friends
d) With family on weekends
e) While travelling
f) In break time
g) other

6) Reason for eating snack
a) I feel Hungry
b) Easily available
c) I have it with friends
d) Convenient
e) Its tasty
f) It is cheap
g) I feel like
h) other

7) Where you generally prefer to eat snack?

a) At home
b) work place/ college/ class
c) At street vendors / tapari/ stall
d) in malls/shops/ restaurants
e) At canteen
f) other

8) What are the factors that you consider while choosing a snack

a) Price
b) Brand
c) Taste
d) Color
e) Hunger
f) Advertisement
g) Friends choice
h) Availability
i) Nutrients
j) Others
k) Habit
l) Ingrdients

9)

Which snacks do you like to buy frequently	What is your weekly expenditure on snacks?
Fruits	
Fried	
Bakery	
Burgers/Sandwiches	
Chaat	
Sweets	
Chocolates	
Packets- Booby/Kurkure/chips	
Biscuits	
Maggi/ other	
Others	

10) Do you read food label before you eat any snack? a) Yes b) No

11) What do you check on the label?

a) Price
b) Expiry
c) Brand name
d) Free gifts
e) Sugar content
f) Salt content
g) Fat content
h) Other

12) Do you eat a snack when you feel stressed, unhappy, angry or bored?
a) Never b) Rarely c) Often

13) Do you skip meal due to snacking? a) Yes b) No

14) which regular meal do you skip due to snacking? a) Yes b) No
breakfast/ Lunch/ Dinner?

15) How often you skip the meal? a) Many times b) Sometimes

16) Do you have snack when you skip any meal? a) Yes b) No

17) How many times in a month do you go out with your family to eat in a restaurant?

18) Which type of food do you have a) Full meal b) Snacks

19) Approximate expenditure/ week or month

20) Preferred type of food (Punjabi/ Chinese/ Italian/ Continental/ Maharashrian)

21) With whom do you generally have food in the restaurant

a) With family
b) Friends
c) Office colleagues
d) Other

Reason for having outside food

a) As a routine practice
b) Corporate meetings
c) Informal party
d) Get Bored
e) Like to have
f) Mood
g) Guests
h) Other

18) Do you feel eating snack food is

a) Healthy
b) Unhealthy
c) Necessary
d) Optional
e) Don’t know

For adolescents and adults

13) Are you following any specific diet? a) Yes b) No

14) If yes which diet

a) Weight loss
b) High protein diet
c) Anti-hypertensive
d) Anti-diabetic

For children/ Adolescent questionnaire

1) Do you get your lunch box from home a) Yes b) No

2) Do you get pocket money from parents? a) Yes b) No

3) How much pocket money do you spend on snack? / month

3) Do your parents get snack at home? a) Yes b) No

4) If yes which snacks do they get often

a) Fruits
b) Fried
c) Bakery
d) Burgers/Sandwiches
e) Chaat
f) Sweets
g) Chocolates
h) Packets- Bobby/Kurkure/chips
i) Chivda/ Chakli
j) Biscuits
k) Aerated drinks
l) Maggie/ noodles
m) Others

5) Are you staying in hostel/ living in campus/ living with parents?

6) What kind of snack food is available near your school/ college?

a) Fruits
b) Fried
c) Bakery
d) Burgers/Sandwiches
e) Chaat
f) Sweets
g) Chocolates
h) Packets- Bobby/Kurkure/chips
i) Biscuits
j) Maggie/ noodles
k) Others

Questionnaire for Adults:

a) Do you have Diabetes? Yes b) No
If Yes, for how many years? ______
b) Do you have hypertension? a) Yes b) No
If Yes, for how many years? ______

5) Anthropometric measurements

Date:

Name of participant:

	Set 1	Set 2
Height (cm)		
Weight (kg)		
Waist circumference (cm)		

Measured by Name:

6) Household questionnaire:

Points for Focused Group Discussion-

**Name of Food**	**Number purchased / week or Kg/month**	**Expenses/ week or month**	**Source- Home/ Outside**
1. Cereals			
2. Dals/Pulses			
3. Other Vegetables			
4. Fruits			
5. Vitamin A rich Fruits and Vegetables			
6. Vitamin C rich Fruits and Vegetables			
7. Green leafy vegetables			
8. Milk and Milk products (low fat) (Milk, Curd, Buttermilk, Paneer)			
9. Eggs			
10. Flesh foods (Meat, Fish, Poultry, Red meat)			
11. Protein powder/ Supplements			
12. Beverages (Tea/Coffee)			
13. Homemade snacks (Breakfast snacks- Pohe, Upma)			
14. Homemade fried snacks (Chakli, Shankar pale, Chiwda)			
15. Homemade sweet snacks (Laddu, Kheer, Basundi, Puranpoli)			
16. Bakery products (Pattice, Khari, Nankatai)			
17. Outside packaged salty snacks (Bobby, Chips, Kurkure)			
18. Outside fried snacks (Batatawada, Samosa.)			
19. Outside sweet Snacks (Mithai, Ice-cream)			
20. Chocolates (Jelly sweets, Cadbury)			
21. Outside Chinese/ Punjabi food/ fast foods (PavBhaji, Pizza)			
22. Sugar-sweetened beverages (Aerated drinks…)			
23. Oil			
24. Sugar			
25. Salt			
26. Ground nuts			
27. Coconut			

7) Focused Group Discussions with key informants with respect to following points-

- Food system in the village or nearby to village like Ration shops/ Grocery shops/ Farms/ Bakery shops
- No of hotels/ restaurants/ fast food center in the village as a sources of Snack Food
- Locally available vegetables and fruits vendors/ market access
- Locally available dairy shops and poultry farms.
- Locally available snack food and no. of snack vendors in the village or near schools/ colleges
- Locally available brands of snack food/ types/ size of packets/ most commonly consumed snacks.
- Any physical activity centers in village or nearby (within distance of around 5km)like Parks, Gym, Yoga center, Play grounds for children, Sports clubs will be documented.
- An attempt will be made to understand respondents view regarding how price elasticity and other drivers will affect their food choices.
- General awareness of localites regarding healthy and non-healthy eating habits will be recorded.
